# The Metropolitan Hospital Sunday Fund

**Published:** 1902-04-26

**Authors:** 


### The Institutional Workshop.

### THE METROPOLITAN HOSPITAL SUNDAY FUND.

### COUNCIL MEETING.

Two important matters were before the Council of this Fund on Thursday, April 17th, viz., the appointment of an honorary secretary, *vice* Sir E. Hay Currie, appointed secretary, and the question of special effort with regard to Hospital Sunday this year. The chairman was the Lord Mayor, who had on his left Sir Edmund Hay Currie, and on his right Mr. Richard Biddulph Martin (hon. secretary), and the Lord Bishop of London. About a dozen members of the Council were present, and letters of regret had been received from Sir Sidney H. Waterlow (vice-president), the Chief Rabbi, Dr. Kitto, and several other council members.

It will be remembered that Mr. Henry N. Custance has been obliged to retire from the office of Secretary to the Fund, owing to continued ill-health, and the work has latterly been done by Sir Edmund Hay Currie, hon. secretary. The Lord Mayor in his opening speech explained that it was understood at the last Council meeting that Mr. Custance would continue in office until the 1st of May; a letter had, however, been received from him, and also one from his medical man, which left no doubt whatever that he would not be able to carry on the work. It was at the Lord Mayor's suggestion that Sir Edmund Hay Currie was approached on the matter, and he took the responsibility of having done so. They had worked together years ago, and he thought that if the whole Metropolis had been searched, a more truly representative secretary could not have been found. He was convinced that Sir Edmund Hay Currie was second to none, and that they could not have made a better choice.

The Bishop of London asked whether the Council ought to confirm the appointment, and the Chairman said that although a free hand had been given, he should like it to be confirmed; so the Bishop proposed, and Mr. Martin seconded, that the appointment be confirmed, and the Council was unanimous in approving it.

The new Secretary said the Council might be quite sure that he would do all in his power for the benefit of the Fund. He had just received a letter from Mr. Custance, thanking the Council for accepting his resignation, and for voting him a pension of £250 after his 28 years of service. Mr. Custance was very glad to hear of the appointment of Sir Edmund Hay Currie. This letter, the Council resolved, should be placed upon the minutes.

The next thing was to appoint a successor to Sir Edmund Hay Currie as honorary secretary, and the Lord Mayor suggested the name of Mr. J. S. Gilliatt, a past governor, and now a director, of the Bank of England, and a merchant well known and respected in the city. His interest in church matters was great, and inasmuch as the Fund had its banking account at the Bank of England, it would be advisable to be in communication with it through one of the directors. He merely threw this out as a suggestion, and left it to others to formally move it.

Mr. Martin moved it, and Sir Henry C. Burdett seconded; the latter said there was no doubt that confidence was the very foundation of success, and this appointment would only increase the confidence that already existed.

#### Suggestions for Hospital Sunday, 1902.

The rest of the time was taken up by a discussion on what special efforts could be made with reference to Hospital Sunday this year, and there seemed to be a general opinion that although much might be done by sermons on the Sunday itself, this should be supplemented by increased personal efforts in the previous week or so, and that, the clergy being busy people with many claims, it would be well if the press would do as they did on a former occasion, and keep the subject before their readers for a week beforehand. This was the suggestion of Sir Henry Burdett.

Mr. Biddulph Martin said that he had often thought that the example of the Dean of Worcester and Canon Fleming might well be followed. Individual touting before the day was the thing. People went out of town for fine Sundays, but if their contributions were secured beforehand, the fund would lose nothing. But it must be done by personal effort. He believed the Lord Mayor had strong views on this point.

#### The Clergy.

The Lord Mayor agreed. The results he said, must be obtained not by expecting people to be in church, but by getting cheques from them beforehand. One who did splendid work in this direction was present, and he hoped they might hear something from him—he alluded to Prebendary Ridgeway. In connection with the coming festivities there would be large undertakings, some of them probably emanating from the Mansion House, but he was anxious that this Fund should not suffer on that account.

Prebendary Ridgeway followed. Eighteen years ago, he said, his hospital collections were £500; they had risen to £1,500. (Applause.) The matter, he went on, was entirely in the hands of the clergy. It was an age of week-ends, and his experience had been that by getting hold of people during the week, and by sending to everybody he knew, not the printed notice issued by the Fund, if he might say so, but a letter written from himself personally, he had been able to raise the large sums which his church had been privileged to send to the Fund. He owned the idea to the suggestion of the Fund in sending its printed letter, but he thought he had improved on it, by sending one in his own hand. This was what wanted bringing home to the clergy. The vast majority did nothing except on the Sunday, and people slipped through a net that had very wide meshes. Until the clergy could be induced to sign the letter, and send it to small givers as well as large, things would remain at a standstill. The jump was taken when a clergyman began to sign the letter himself, in his own hand, and to tell the people it was their duty to give.

Another Clergyman, and one with country experience, said he always sent his district visitors round before the day to collect; the plan had been found very successful.

Then Archdeacon Sinclair proposed a resolution: That it was desirable that a special effort should be made this year to increase the collections, and that the matter should be referred to the General Purposes Committee. He thought the meeting had resulted in very valuable suggestions, which the General Purposes Committee might well embody in a leaflet. Prebendary Ridgeway and Canon Fleming might perhaps be asked to meet the committee and help with further advice. Even in the country much might be done by lady visitors, deaconesses, etc. Certainly the poorer as well as the richer should be encouraged to give, by way of showing gratitude for the blessings of this year, and the approaching end of the war. Perhaps the Lord Mayor could send a special letter from the Mansion House to those clergy who sent no contribution last year. What Prebendary Ridgeway had said was perfectly true; if the clergy depended on the one day very little could be done. On one occasion, when his term of residence at St. Paul's coincided with Hospital Sunday, he sent round beforehand to all the people in the Churchyard. He did not think the parishes were worked properly.

The resolution was supported by several speakers. One clergyman, however, seemed to think that the value of the sermon had been too lightly esteemed. He thought if Sir Edmund Hay Currie was to write to some of the great preachers they would be willing to give a sermon, and to come up for the day to do so. People might write to the Lord Mayor, as he believed some already did, to send them a preacher for the occasion. The speaker was assured that no slight on the sermon had been intended; the suggestions merely guarded against loss to the Fund through people being out of town.

The Laity, Especially the Press.

Sir Henry Burdett, speaking on the report, said he found that although the number of contributing congregations had increased by 75 per cent., the corresponding increase in the amounts was not maintained in the proportion which would be expected. In 1901, 1,820 congregations made collections, with a result of £36,000, which was £4,000 lower than in 1896, when there were only 1,811 contributing congregations. The laity ought to have an immense sympathy with the clergy, especially with those who had large expenses and weak revenues. He thought they should approach the smaller men, with humbler congregations, who needed encouragement just as much. Canon Millar, who had much to do as organiser of the Fund, followed the plan of the "Golden Pheasant Circular." When there was not importance, there were numbers, and "numbers," said the speaker, "do tell up very much." The area of influence must be widened; people must be got hold of who never went to church. A large number of people gave nothing, and it should be pointed out to them that from the point of view of citizenship, as well as from a religious point of view, it was a duty they owed to the State. The press, again, could help to a very great extent. What was wanted this year, more than ever before, was a special effort on their part; they must begin to help the Fund on the Monday before Hospital Sunday and go on every day of that week. They did this once before, in the year of the Fund's greatest success (Diamond Jubilee year), and if this was done again, and the clergy at the same time were dinning it into the ears of their people, a result would be brought about which could be obtained in no other way, and probably a sum of £60,000 might be raised.

Going for a Record.

The Lord Mayor said the clergy did yeoman's work on Hospital Sunday; he was not, however, going to be satisfied with the sum named by the last speaker. He meant this to be a record year, and he aimed at £100,000. The laymen ought to come in and help. Methods of going to work would be discussed by the General Purposes Committee. In concluding his speech the Lord Mayor said "we are going for a record this year, and all must help. The Church militant has many armies and corps."

The resolution was carried with acclamation.

Anti-Vivisectors.

Several speakers thought the distribution of anti-vivisection literature at church doors, especially when coupled with the name of one of the Council, should be put a stop to. Dr. Glover advised that it be left severely alone; people would learn that, if experiments were conducted humanely, the animals had their part to play in the study of disease.

Sir Henry Burdett and others thought the gentleman referred to, whose name appeared on the leaflets, should be written to, and the Lord Mayor promised that it should have the attention of the General Purposes Committee.

In proposing a vote of thanks to the Chair, the Lord Bishop of London said the poor clergy got so many circulars asking them to support this fund and that, that perhaps too much was sometimes expected of them.

Sir Joseph Savory seconded, and the Lord Mayor again expressed his hope that his year of office might be signalised by a record collection for the Fund.

